# Identification of an Astrovirus Commonly Infecting Laboratory Mice in the US and Japan

**DOI:** 10.1371/journal.pone.0066937

**Published:** 2013-06-25

**Authors:** Terry Fei Fan Ng, Nikola O. Kondov, Nobuhito Hayashimoto, Ritsuki Uchida, Yunhee Cha, Ashley I. Beyer, Walt Wong, Patricia A. Pesavento, Hiroshi Suemizu, Marcus O. Muench, Eric Delwart

**Affiliations:** 1 Blood Systems Research Institute, San Francisco, California, United States of America; 2 Department of Laboratory Medicine, University of California San Francisco, San Francisco, California, United States of America; 3 ICLAS Monitoring Center, Central Institute for Experimental Animals, Kawasaki, Japan; 4 Department of Pathology, Microbiology and Immunology, University of California Davis, Davis, California, United States of America; 5 Biomedical Research Department, Central Institute for Experimental Animals, Kawasaki, Japan; Centers for Disease Control and Prevention, United States of America

## Abstract

Mice (*Mus musculus*) are the most commonly used laboratory animals. Viral metagenomics on tissues of immunodeficient mice revealed sequences of a novel mammalian astrovirus. Using PCR, we screened mice from 4 breeders, 4 pharmaceutical companies, 14 research institutes and 30 universities in the US and Japan. Mice from one US breeder tested positive while none from Japanese breeders were positive for MuAstV. Mice in over half of the universities (19/30), institutes (7/14) and pharmaceutical animal facilities (2/4) investigated revealed the presence of MuAstV. Nine mice strains tested positive including both immunodeficient strains (NSG, NOD-SCID, NSG-3GS, C57BL6-*Timp-3* ^−/−^, and uPA-NOG) and immunocompetent strains (B6J, ICR, Bash2, BALB/c). Our data indicates that MuAstV has a wide geographical, institutional and host strain distribution. Comparison of the MuAstV RdRp sequences showed numerous mutations indicating ongoing viral divergence in different facilities. This study demonstrates the need for metagenomic screening of laboratory animals to identify adventitious infections that may affect experimental outcomes.

## Introduction

Mice play a significant role in biomedical research and are used to study basic biological mechanisms, model diseases and test new therapies [1]–[3]. Commercial mouse strains encompass a wide range of genotypes and phenotypes. Various outbred and inbred mouse strains are used in research as well as an ever-increasing number of genetically modified strains used to study the contribution of specific genes. For instance, numerous immunocompromised laboratory mouse strains have been developed that are deficient in various components of the innate or adaptive immune response. Severely immunodeficient mice, in particular, have proven useful for creating in vivo models for the study of human disease [4]–[7]. Elimination of the adaptive immune response in mice allows for the engraftment of human cells and tissues [4]–[7]. The resulting “humanized” mice serve as model organisms for a variety of disorders and for pre-clinical research [1], [3], [6], [7]. Introduction of hematopoietic stem cells into immunodeficient mice, for example, allows for the in vivo study of their differentiation into the various components of human blood [7]–[11]. Humanized mice have aided in the development of gene therapies and cell-based therapies for hematopoietic disorders in humans [7], [12]–[26].

Biomedical research using laboratory mice requires a healthy animal colony [27]. Immunocompromised mice are especially susceptible to infections. For example, a murine norovirus associated with encephalitis, meningitis, hepatitis and vasculitis was recently discovered in immunodeficient laboratory mice [28]. Such pathogens can impact biomedical research programs by affecting research outcomes and by increasing the time and cost to rebuild mouse colonies [27].

In order to uncover viruses circulating in laboratory mice, we employed an approach that does not necessitate prior knowledge of virus types. Viral metagenomics, using unbiased amplification of enriched viral particles-associated nucleic acids and next generation sequencing provides an efficient method for characterizing the viruses present based on sequence similarity with any previously characterized viral genome [29]–[31]. This method has been applied in the discovery of viral pathogens associated with infections in humans, as well as in domestic and wild animals [19], [30], [32]–[36].

We performed a viral metagenomic analysis of tissue samples obtained from NOD.Cg-*Prkdc^scid^ Il2rg^tm1Wjl^*/SzJ (NSG) immunodeficient mice. Following the identification of a novel astrovirus, which was also recently described by other groups [24], [37], we used PCR and sequencing to determine the prevalence of this virus in various mouse strains maintained at Blood Systems Research Institute (San Francisco, CA), the Central Institute for Experimental Animals (CIEA; Kawasaki, Japan) as well as other vivaria in Japan, and mice from The Jackson Laboratories (JAX; Bar Harbor, MN and Sacramento, CA).

## Materials and Methods

### Sourcing and Care of Mice

The experiment in the Blood Systems Research Institute (BSRI) was conducted with approval of the Institutional Animal Care and Use Committee at ISIS Services LLC (San Carlos, CA). The experiment in Japan was approved by Institutional Animal Care and Use Committee in the Central Institute for Experimental Animals (CIEA) based on the Regulations for Animal Experimentation of the CIEA. For MuAstV detection in a US breeder, immunodeficient and BALB/c mice were ordered from Jackson Laboratory facility in Sacramento, CA. Upon arrival, fecal samples were collected from their commercial transport container. Becuase the same strain of mice ordered from JAX was held in a single transporting container, each container was considered as one sample to avoid over-estimation of the viral prevalence.

All mice housed in the BSRI originated from JAX or CIEA. Mice that have been bred or housed at BSRI are described as originating from BSRI. The mouse vivarium at BSRI is a small facility with access restricted to trained and authorized personnel. Throughout this study, the vivarium operated at approximately half of a full occupancy of 504 cages. All mice were housed in laminar flow racks within sterile disposable microisolator cages (Innovive Inc., San Diego, CA). All cage bedding, environmental enrichment, food and water was radiation sterilized. All husbandry and experimental procedures were performed within a laminar-flow biosafety cabinet or cage-changing station. During routine changes in caging, mice are transferred to clean cages using forceps that are rotated through a disinfectant solution (Vimoba, Quip Labs, Wilmington, DE) and 70% ethanol to minimize the risk of pathogen transfer among cages. Sentinel mice exposed to soiled bedding were used to screen for recognized murine pathogens.

The two NSG mice used for metagenomic analysis were raised from a colony maintained at BSRI. They were evaluated for the presence of any underlying viral infection. By all appearance and behavior, the animals appeared to be healthy. Histological analysis was performed to assess the healthy status of the mice. A full necropsy was performed and tissues were fixed in 10% buffered formalin. The collection protocol included sections of cerebrum, cerebellum, trigeminal nerve, skeletal muscle, salivary gland, eye, ear, lung, heart, liver, spleen, duodenum, pancreas, jejunum, colon, lymph nodes (peripheral, mesenteric), liver, kidney, adrenal, and skin. Formalin-fixed tissues were processed and embedded in paraffin, sectioned at 5 µm, and stained with hematoxylin-eosin (H&E) for histologic evaluation.

The colony of NSG mice was founded with breeding pairs obtained from JAX 35 months before. All other mice in the vivarium were obtained from JAX with the exception of breeding pairs of NOD.Cg-*Prkdc^scid^ Il2rg^tm1sug^ Tg(Alb-Plau)11-4*/ShiJic (uPA-NOG) mice [38] obtained from CIEA and C57BL6-*Timp-3* ^−/−^ obtained from the University of Texas Health Science Center-Houston. During the last year the only viral infection detected in the vivarium by the sentinel program was a Murine Norovirus-1 infection associated with a colony of C57BL6-*Timp-3* ^−/−^ mice, which appeared to resolve upon rescreening several months later.

All cecum samples from mice in Japan were collected during their microbiologic monitoring tests from Nov. to Dec. 2012. Due to service contracts, the names of the institutes were made anonymous, and the breeder origins of the mice were not disclosed. Animals were euthanized by exsanguination from the axillary artery and vein under isoflurane anesthesia. Necropsy was then performed, and nucleic acids from the cecum samples were extracted.

### Viral Metagenomic Sequencing

Two NSG immunodeficient mice from BSRI of approximately five weeks of age were sacrificed by CO_2_ asphyxiation, and their internal organs (lung, spleen, bone marrow, liver and brain) dissected. The organs were pooled for metagenomic analysis. Viral purification from the pooled organs was carried out by homogenization, freezing and thawing, filtration and nuclease treatment of filtrates using previously published protocols [19], [31]. Viral nucleic acids were used in reverse transcription (RT) reactions with a primer comprising of 20-base oligonucleotide followed by a randomized octamer sequence at the 3′ end [19], [31]. After denaturation the cDNA was amplified by a single round of DNA synthesis using Klenow fragment polymerase (New England Biolabs). Random PCR was performed using a primer consisting of only the 20-base fixed portion of the random primer. This library was sequenced with 454 pyrosequencing using GS FLX+ platform. A total of 4,500 reads were generated from two animals. Sequences were trimmed of primer sequences and those sharing at least 95% nucleotide identities over 35 bases were assembled into contigs. Assembled contigs and singlets were compared to the GenBank non-redundant nucleotide and protein database using BLASTx with an E-value cutoff of 10^−4^ [31].

### PCR and Genome Sequencing

The partial genome of the murine astrovirus BSRI (MuAstV BSRI) was obtained by Sanger dideoxy sequencing of PCR products obtained using combinations of primers to connect the metagenomics-derived genome fragments. Primers were designed using PRIMER3 [20], [39]. Using the cDNA derived from the original mice, PCRs were performed with LA Taq (Clontech) according to the manufacturer’s instructions. PCR reactions were carried out with a “universal touch-down PCR” suitable for the melting temperatures of all primers as follows: 95°C for 5 min, 45 cycles of [94°C for 1 min, 58°C minus 0.2°C per cycle for 1 min, 72°C for 1 to 5 min], followed by 72°C for 10 min. The partial genome was extended using 5′ and 3′ RACE amplification kits (Invitrogen) according to the manufacturer’s instructions and previously described protocols [31].

### Phylogenetic Analyses

To determine the relationship of murine astrovirus BSRI to other astroviruses, two phylograms were created based on the nucleotide and the amino acid sequences encoded by the RNA dependent RNA polymerase (RdRP) gene. The sequences were aligned using Mafft 5.8 [21] with previously described parameters [31], [34]. For amino acid alignment, maximum likelihood (ML) trees were generated from the alignment using RAxML, where Dayhoff similarity matrix parameters were used specifying a general time reversible model with a gamma distribution for rates over sites [22]. ML trees were run with 100 bootstrap replications and resulting trees were examined for consistency with published phylogenetic trees. For nucleotide alignment, neighbor joining tree was generated using MEGA [23] using the *p*-distance model with 10,000 bootstraps performed. Mid-point rooting was conducted using MEGA [23].

### Specific Reverse Transcriptase PCR (RT-PCR) for Murine Astroviruses

RNA was extracted from mice livers and feces using the QIAamp MinElute Virus Spin kit (Qiagen). cDNA was generated from the sample RNA using the SuperScript III reverse transcriptase (RT; Invitrogen) with 100 pmol of random hexamer primer, 10 pmol of each dNTP, 10 µL of RNA, 1 µL buffer, 5 mM DTT, 1 µL of RiboLock RNase Inhibitor (Fermentas), and 200 units of RT enzyme following the manufacturer’s instruction.

To screen for MuAstV, primers MuAstV-AF (5′ GCACACGTAGTTGGGAGTGA 3′) and MuAstV-AR (5′ TGGTGTGTATCCCAAGGACA 3′) were used in PCR reactions targeting 328 bases of the ORF1a. Sample tested positive was re-confirmed by another PCR, using primers MuAstV-BF (5′ GAATTTGACTGGACACGCTTTGA 3′) and MuAstV-BR (5′ GGTTTAACCCACATGCCAAA 3′) targeting the RdRP, producing an amplicon of 328 bases. The PCR reactions were carried out using the touch-down PCR conditions described above, using LA taq, EX taq (Clontech) or equivalent, except that the cycle extension time used was 1 min. Amplicons were analyzed by ethidium bromide gel electrophoresis and sequenced using Sanger dideoxy sequencing.

## Results

Viral metagenomic was performed on pooled tissues from two NSG immunodeficient mice approximately five weeks old. All tissues examined were histologically normal with no detectable inflammation. An initial database search using 4500 sequence reads using BLASTx in June 2012 indicated that nearly half of the sequences (n = 2035) originated from a novel astrovirus with ∼ 60% protein sequence identity to human and porcine astroviruses. A subsequent search with an updated GenBank database (Sep 2012) revealed the sequences were closely related to the murine astrovirus (MuAstV) reported by two groups in late 2012 [24], [37]. No other viral sequences were identified in these two laboratory mice.

**Figure 1:**
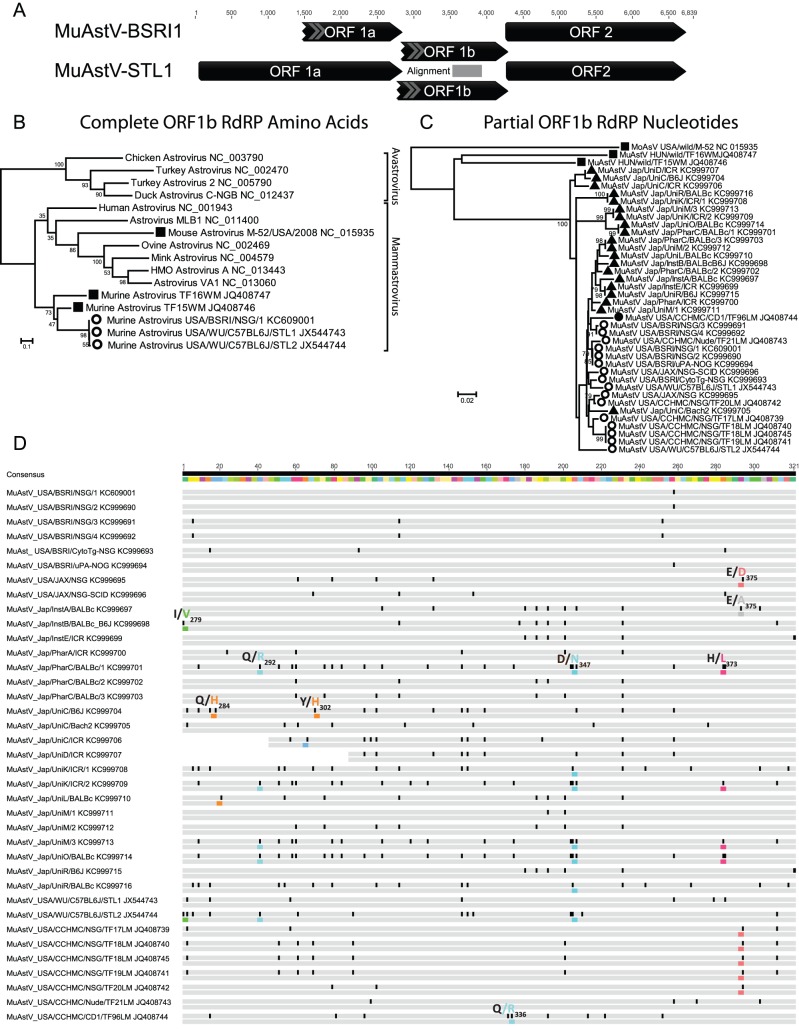
Genome organization and phylogenetic analyses of the murine astrovirus. A) Genome organization of MuAstV. B) Phylogenetic analysis of the translated RdRP sequence of the murine astrovirus USA/BSRI/NSG/1 and representatives of the *Astroviridae* using the maximum likelihood method. C) Phylogenetic analysis of the RdRP nucleotide sequence of the murine astrovirus in laboratory mice from the USA and Japan, along with murine astrovirus in wild and laboratory mice from the Genbank database. Sequences from wild mice were labeled with squares; laboratory mice from USA with circles; laboratory mice from Japan with triangles; immunocompetent mice were labeled black and immunodeficient mice were labeled white. D) Comparison of nucleotide sequences of MuAstV in this study with published sequences in GenBank. For each sequence, the first row denotes nucleotides in which discrepancies were highlighted in black. The second row denotes amino acids in which discrepancies were highlighted in color with a note – consensus amino acid was followed by the mutated amino acid and the position corresponded to the translated ORF1b RdRP gene.

A partial genome of MuAstV-BSRI1 (Genbank Accession KC609001), of 5274 bases was characterized using PCR and rapid amplification of cDNA ends followed by Sanger sequencing. MuAstV genome contained three overlapping open reading frames (ORF1a, ORF1b, and ORF2). ORF 1a, which encodes for protease, was partially sequenced (1354 bases). ORF1b and ORF2, which encodes the RNA-dependent RNA polymerase (RdRP) and capsid respectively, were completely sequenced (1351 and 2789 bases). MuAstV-BSRI1 shared 94% nucleotide identities with the MuAstV genomes published in late 2012 by two separate groups [24], [37]. Phylogenetic analysis of the translated RdRP sequence further confirmed that the murine astrovirus in this study belonged to the same species as the recently described murine astroviruses [24], [37], belonging to the third genogroup of *Mammastrovirus* (Fig. 1).

Using PCR, animals from multiple breeders, research institutes and universities from the USA and Japan were screened for MuAstV. In the USA, murine astrovirus was detected in young adult mice shipped from the Jackson Laboratory in Sacramento, CA and at BSRI (Table 1). Fecal samples from immunodeficient NSG and NOD.CB17-*Prkdc^scid^*/J (NOD-SCID) mice testing immediately upon arrival from the Jackson Laboratories tested positive for MuAstV while feces from BALB/c mice were PCR negative. From BSRI raised mice, MuAstV was present in the feces of 100% (6/6) of the immunocompromised mice tested, and 0% (0/7) of the immunocompetent mice (Table 1). The absence of MuAstV in immune-competent mice in the US might be due to the small sample size, and that most of the mice maintained at BSRI are adults that may have cleared their infections. Both young and old adult immunodeficient mice tested positive for the virus. Fecal samples from uPA-NOG mice originating from CIEA, Japan but raised in a breeding colony at BSRI tested MuAstV positive.

**Table 1:** PCR prevalence of MuAstV in US facilities in liver and feces samples.

	Sample		Feces		Liver	
Hosting facility	Strain^1^	Age (days)	# of Positive	# of Tested	# of Positive	# of Tested
BSRI	BaLB/cJ	211/246			0	2
	CByJ.B6-Tg(UBC-GFP)30Sha/J	533			0	2
	C57BL/6-Tg-(UBC-GFP)30Sha/J	206/385			0	2
	C57BL/6J	68/411			0	1
	C57BL6-Timp-3−/−	129			1	1
	NSG	45/116/242			3	3
	NSG-3GS	92			1	1
	uPA-NOG	199–245 (pooled)	1	1		
The Jackson Laboratory	BaLB/cJ	44	0	1		
	NSG	37	1	1		
	NOD-SCID	37	1	1		

1 Strain abbreviations used: NOD.Cg-*Prkdc^scid^ Il2rg^tm1Wjl^* Tg(CMV-IL3,CSF2,KITLG)1Eav/MloySzJ (NSG-3GS), NOD.Cg-*Prkdc^scid^ Il2rg^tm1Wjl^*/SzJ (NSG), NOD.Cg-*Prkdc^scid^ Il2rg^tm1sug^ Tg(Alb-Plau)11-4*/ShiJic (uPA-NOG), and NOD.CB17-*Prkdc^scid^*/J (NOD-SCID).

In Japan, cecum samples from laboratory mice from 3 breeders, 4 pharmaceutical companies, 13 research institutes and 30 universities were screened for MuAstV (Table 2). All three Japanese breeders tested negative in all samples investigated. Mice from two out of four pharmaceutical companies tested positive for MuAstV. Seven out of thirteen research institutes showed positive results in the MuAstV PCR tests, while murine cecum samples from 17 out of 25 of the universities tested positive.

**Table 2:** PCR prevalence of MuAstV in Japanese facilities in cecum sample.

Hosting facility	Strain	# of Positive	# of Tested	Percentage Positive
**Breeder A**	B6J		8	0%
	IQI		14	0%
	mdx		2	0%
**Breeder B**	B6J		8	0%
	BALB/c		5	0%
	ICR		12	0%
	NOD-scid		10	0%
**Breeder C**	ICR		2	0%
	NOD-scid		2	0%
**Institute A**	BALB/c	1	9	11%
	ICR		6	0%
**Institute B**	B6J	1	4	25%
	BALB/c×B6J	1	1	100%
**Institute C**	ICR		5	0%
**Institute D**	ICR	1	5	20%
**Institute E**	ICR	1	5	20%
**Institute F**	ICR		10	0%
**Institute G**	ICR		5	0%
**Institute H**	B6J		1	0%
	ICR	2	7	29%
**Institute I**	B6J		2	0%
	BALB/c		3	0%
**Institute J**	ICR	2	2	100%
**Institute K**	ICR	1	1	100%
**Institute L**	ICR		1	0%
**Institute M**	unknown		1	0%
**Pharmaceutical A**	ICR	1	2	50%
**Pharmaceutical B**	ICR		2	0%
**Pharmaceutical C**	BALB/c	3	9	33%
**Pharmaceutical E**	BALB/c		1	0%
	ICR		5	0%
**University A**	ICR		5	0%
**University B**	ICR		5	0%
**University C**	Bach2	1	1	100%
	Gfi1/CD4-cre		1	0%
	ICR	1	2	50%
	Menin		1	0%
**University D**	ICR	1	5	20%
**University E**	B6J		3	0%
	ICR	3	11	27%
	PKA		1	0%
**University F**	ICR	2	7	29%
	unknown		1	0%
**University G**	ICR		2	0%
**University H**	ICR		4	0%
**University I**	ICR		4	0%
**University J**	ICR		4	0%
**University K**	ICR	2	9	22%
**University L**	BALB/c	1	2	50%
**University M**	unknown	3	10	30%
**University O**	BALB/c	1	5	20%
	ICR	1	5	20%
**University P**	ICR		3	0%
**University Q**	ICR		6	0%
**University R**	B6J	2	3	67%
	BALB/c	1	1	100%
	ICR	4	11	36%
**University S**	B6J		1	0%
	ICR	2	3	67%
**University T**	B6J		1	0%
	ICR	1	1	100%
	unknown	1	1	100%
**University U**	ICR	1	3	33%
**University V**	B6J		2	0%
	ICR	1	5	20%
**University W**	B6J		1	0%
**University X**	B6J	1	1	100%
	B6N		1	0%
	ICR		5	0%
	unknown	2	3	67%
**University Y**	ICR		1	0%
**University Z**	B6J	1	1	100%
**University AA**	B6J		1	0%
	BALB/c		1	0%
	C3H	1	1	100%
**University AB**	C3H		1	0%
	ICR	1	1	100%
**University AC**	BALB/c	1	1	100%
**University AD**	ICR		2	0%
**University AE**	B6J		1	0%
	ICR	1	2	50%

Laboratory mice stains testing positive in the US samples were immunodeficient NSG, NOD-SCID, NSG-3GS, C57BL6-*Timp-3* ^−/−^, and uPA-NOG mice. Stains positive in Japan were all immunocompetent B6J, ICR, Bash2, BALB/c mice, since immunodeficient mice investigated were from breeders and were all negative. Higher sample size (n>10) was collected for five mouse strains in Japan, namely B6J, BALB/c, ICR, IQI and NOD-SCID (Table 3). MuAstV was detected in 13%, 22% and 16% of the B6J, BALB/c, ICR strains respectively. No Japanese samples from IQI and NOD-SCID mice tested positive.

**Table 3:** Percentage of MuAstV PCR positives in cecum samples from five different mice strains (n>10) in Japan facilities.

Strain	# of Positive	# of Tested	Percentage
B6J	5	38	13%
BALB/c	8	37	22%
ICR	29	176	16%
IQI	0	14	0%
NOD-SCID	0	12	0%

All MuAstV detected by PCR in US and Japanese laboratories were closely related phylogenetically (Fig. 1B and C) with less than 10% nucleotide sequence divergence (Fig.1D). In contrast, MuAstV from laboratory mice is divergent to other MuAstV identified in wild mice [37], with sequence divergence ranging between 26–33% (Fig. 1B and C).

Mutation sites on the laboratory mice MuAstV RdRP fragment (321 bases) used for the diagnostic PCR were analyzed (Fig. 1D). Synonymous mutations were frequent and, in some cases, common mutations were seen among mice of the same strain in the same facilities (between MuAstV USA/BSRI/NSG/1 and 2; between MuAstV USA/BSRI/NSG/3 and 4; MuAstV USA/CCHMC/NSG/TF18LM and 19LM) (Fig 1D). Furthermore, mice of the same strains maintained in different facilities contained different MuAstV mutations, for example, NSG mice in BSRI differed from those in CCHMC. Out of the 107 codons RdRP sequence analyzed, 8 (7.5%) non-synonymous mutation sites were recognized (Fig. 1D). The most common NS mutations were 292Q>R and 347D>N mutations, both of which were found in mice from the US and Japan. The 373H>L mutation only occurred in Japan and the 375E>D mutation only in mice from the US.

## Discussion

We identified a murine astrovirus (MuAstV) using a metagenomic approach in pooled tissues from immunodeficient laboratory mice. PCR screening revealed that MuAstV is commonly found in mice facilities in the USA and Japan, including breeding facilities, universities and research institutes. MuAstV was detected in a variety of mouse strains, most consistently in strains with compromised immune systems (NSG, NOD-SCID, NSG-3GS, C57BL6-*Timp-3* ^−/−^ and uPA-NOG), but also in some mouse strains with functional immune systems (B6J, ICR, Bash2, and BALB/c).

We also investigated MuAstV infections in facilities that maintain both immunodeficient and immunocompetent mice, including three Japanese breeding facilities and BSRI (Table 1 and 2). The three Japanese breeding facilities were free of MuAstV. At BSRI, MuAstV was detected in all immunocompromised mice tested but none of the immunocompetent mice. A likely explanation is that both immunodeficient and immunocompetent mice are susceptible to MuAstV, but adaptive immunity is required to clear the virus [24]. No inflammation was detected in the MuAstV-positive immunodeficient NSG mice by histologic evaluation; however, the immunodeficiency of the mice may actually mask the inflammatory response to some degree. In asymptomatic mice with or without immunity, Yokoyama et al [24] detected high viral load (up to 10^9^ genome copies per fecal pellet). The same study detected MuAstV in liver and kidney tissues of immunocompromised mice, but not in those internal tissues of immunocompetent mice, suggesting MuAstV infects immune-compromised mice systemically. Comparatively, human astroviruses are generally associated with gastroenteritic symptoms in patients with a weakened immune system [15], [40]. For instance, HIV-positive individuals, young children, and the elderly are especially sensitive to enteric astrovirus infection [26], [40], [41]. Young children infected with human astrovirus are typically able to clear the virus within two weeks [42]. We hypothesize that the virus preferentially infects young mice, perhaps while still in the nest, but that infection is then cleared only in mice with functional immune systems.

MuAstV can clearly infect both immunocompetent and immunodeficient mice, and its prevalence is likely to be determined by a number of factors: 1) whether the mice were provided by a breeder that has a history of MuAstV; 2) whether the mice were held in a facility with other MuAstV positive mice; and 3) whether the mice can clear the virus with a functional immune system, and have enough time to do so (by aging). Despite the small sample size, all immunodeficient mice in BSRI were MuAstV positive, possibly because founder mice arrived infected from the US breeder JAX. On the contrary, all Japanese immunodeficient mice tested negative for MuAstV, in which the two breeders were MuAstV-free.

MuAstV strains in laboratory mice were all closely related (Fig. 1), including mice maintained in the USA and Japan. Synonymous mutations occurred in all laboratory mice (Fig. 1D), and some mutations were identical in mice of the same strain in the same university. Certain non-synonymous mutations (347D>N) were found in different strains in different facilities possibly reflecting convergent evolution. We do not observe any significant patterns in MuAstV mutations between the outbred (ICR) or inbred derived (B6J) host strains. Since laboratory mice are bred from existing colonies with no or limited contact with wild mice, it is possible that the current MuAstV diversity in laboratory mice is the result of a single, or limited, incident of astrovirus infections in ancestral laboratory mouse populations that has survived undetected in research facilities.

While very closely related to each other in the sequenced RdRP region (0–9% nucleotide divergence, Fig. 1C and D), the MuAstV sequences from laboratory mice differed from the two previously described wild MuAstV species described in Hungary by 26–33% and the mouse astrovirus (MoAsV) in USA by 43–45%. The three wild mouse astroviruses were highly distinct from one another differing in RdRP by 42–45% [37], [43] (Fig. 1B and C). As was seen with the multiple astroviruses recently identified in other host species such as humans [44], pigs [45], and Californian sea lions [46] it is likely that yet more astrovirus species remain to be characterized in wild mice.

The discovery of MuAstV in laboratory mice could have implications for research using mice, since as many as 9 strains of laboratory mice were positive for MuAstV in facilities in two countries and more than half of the institutes or universities investigated in this study tested positive for MuAstV in some of their mice (Table 1 and 2). For those strains where larger sample size was tested, the prevalence of MuAstV ranged from 0% to 22% (Table 3). We therefore anticipate that other mice facilities are also contaminated with MuAstV. Although MuAstV infected immunodeficient mice showed no sign of pathology using histopathological microscopy, virus replication may incur fitness cost and distress or otherwise affect immunological reactions. The effects of co-infections including MuAstV are also not known, as are the consequences of MuAstV in different immunodeficient strains. In studies using mice as models for cancer, autoimmune and infectious diseases, the presence of MuAstV might affect outcomes and the interpretation of laboratory results. Since MuAstV infects immunocompromised mice readily and chronically [24], it may also be useful as an animal model for the investigation of astrovirus infections.

This study demonstrates the utility of metagenomic analyses in identifying previously unrecognized viral infections in laboratory animals. The same MuAstV was recently characterized in an animal facility at the University of Cincinnati, OH, USA by Frakas et al using a consensus PCR approach [37] and at Washington University MO, USA by Yokoyama et al using a metagenomics approach [24] similar to that used here in both immunodeficient and immunocompetent laboratory mice as well as from three commercial vendors in the USA [24] indicating a wide distribution in North American laboratories. This study supplements previous studies by demonstrating a wider presence of MuAstV in many strains, facilities and geographical regions (US and Japan), and by showing viral sequence divergence in different facilities worldwide.

While no other viral sequences were observed in the two laboratory mice tissue virome, further studies of rodents and other laboratory animals may reveal the presence of more unsuspected viral infections underlining the need for continuous metagenomic screening particularly of immunodeficient animals to ensure their wellness as well as the accuracy and reproducibility of biomedical research using animals.
